# Effect of a health education program on type 2 diabetes prevention among high school teachers in Erbil, Iraq

**DOI:** 10.3389/fpubh.2026.1818180

**Published:** 2026-06-19

**Authors:** Shalaw Faris Ahmed, Kareem Fattah Aziz

**Affiliations:** Community Health Nursing Department, College of Nursing, Hawler Medical University, Erbil, Iraq

**Keywords:** behavioral change, health education program, high school teachers, Iraq, metabolic improvement, preventive practices, public health intervention, type 2 diabetes

## Abstract

**Background and aim:**

In Erbil, Iraq, the rising burden of type 2 diabetes (T2D) calls for effective preventive strategies targeting modifiable behaviors. This study aimed to evaluate the effect of a structured health education program on preventive practices and metabolic improvements related to T2D among high school teachers in Erbil.

**Method:**

This quasi-experimental study was conducted from October 27, 2024, to June 1, 2025, in five major high schools in Erbil, using purposive sampling method. Data were collected using a self-structured questionnaire assessing sociodemographic characteristics and 15 preventive practices and metabolic improvement related to T2D. Paired sample *t*-tests, one-way ANOVA, *post-hoc* Tukey tests, effect sizes, and multiple linear regression analyses were performed using SPSS version 27 (IBM Corp., Armonk, NY), with *p* < 0.05 considered statistically significant.

**Results:**

A total of 102 high school teachers participated in the study, equally divided into intervention and control groups (*n* = 51 each). The health education program led to significant improvements in preventive practices and metabolic improvement, with the overall practice score increasing from 45.08 to 59.58 in the intervention group (*p* < .001), while no significant change was observed in the control group (44.54 to 44.35, *p* = 0.677). Marked behavioral gains included increased HbA1c testing, physical activity, label reading, and reduced sugary beverage intake. Clinical outcomes such as BMI, fasting glucose, and lipid profile also improved significantly post-intervention. A very large effect size was observed for the overall practice score (d = 1.94). Regression analysis identified group assignment, younger age, female gender, and family history of diabetes as significant predictors of improved post-intervention scores.

**Conclusions:**

Structured quality health education was associated with significant improvement in preventive practices and metabolic risk reduction measures that translated to favorable clinical outcomes among high school teachers. Policymakers and healthcare providers should implement targeted educational programs in schools to enhance health literacy, support behavioral change, and reduce the burden of type 2 diabetes in at-risk populations.

## 1 Introduction

Type 2 diabetes (T2D) is a chronic metabolic disorder marked by elevated blood glucose levels due to insulin resistance and progressive β-cell dysfunction (1, 2). It is a growing global concern and a primary contributor to multiple health complications including cardiovascular diseases and kidney failure. Recent data suggest that T2D now affects individuals across all age groups, although middle-aged adults remain disproportionately burdened by its onset and consequences (3). Globally, an estimated 589 million adults had diabetes in 2024, and nearly 90–95% of all diagnosed cases are T2D (4). The growing epidemic of T2D and its burden on health care systems point to the need for primary preventive and cost-effective strategies designed to achieve sustainable behavioral changes and promote metabolic health (4).

Given this growing burden, there is an urgent demand for public health interventions aimed at reducing modifiable risk factors of T2D through education and early prevention. The silent and progressive nature of the disease means that individuals are often unaware of the condition until complications arise, necessitating proactive preventive strategies. Although pharmacological treatments remain essential for glycemic control, recent approaches have increasingly emphasized lifestyle-based and community-oriented interventions to prevent or delay disease progression (5, 6). Educational programs targeting knowledge, diet, and physical activity have demonstrated beneficial effects; however, the consistency and long-term sustainability of these outcomes remain variable (7). A community-based approach was selected because behavioral risk factors for type 2 diabetes are influenced not only by individual choices but also by social and workplace environments. High school teachers were considered an appropriate target group because they work within a structured community setting where shared learning, peer interaction, and daily social contact may support the adoption and maintenance of healthy behaviors. In addition, teachers can influence health awareness among students and the wider community through their professional and social roles. Delivering the intervention within the school environment also provided a practical setting for integrating health education into participants' daily routines and encouraging collective participation in lifestyle modification activities.

T2D involves numerous interrelated risk domains, including poor dietary habits, sedentary lifestyle, high stress levels, genetic predisposition, and limited awareness of early warning signs (8). Conventional interventions focusing on a single risk factor often fall short in achieving long-term remission or sustained behavior change. In contrast, comprehensive lifestyle-based approaches that collectively target knowledge, attitudes, and behaviors may support sustainable lifestyle modifications and improved metabolic outcomes (5, 6). By fostering awareness and self-efficacy, these programs may support sustainable lifestyle adjustments that reduce T2D onset and severity. Moreover, teacher-targeted interventions have the potential to create ripple effects by influencing both personal behaviors and those of students and families within their reach (9, 10).

Preliminary studies show that targeted health education can lead to measurable improvements in knowledge, attitudes, and lifestyle practices linked to diabetes prevention and management (11). Long-term, low-intensity participation in Structured Education may lead to improved metabolic outcomes for some patients with early-stage T2D (12). Additionally, interventions aimed at educators have shown promise in increasing awareness of risk factors and encouraging peer-driven dissemination of health knowledge. These outcomes align with the broader goals of preventive health education to embed healthy practices into daily routines and community culture. Such teacher-focused strategies represent a scalable and impactful path to controlling the expanding diabetes epidemic through upstream educational efforts (13).

Additionally, some studies propose that these programs promote personal self-efficacy and confidence in taking up and maintaining healthy lifestyle behaviors and thus increase their capacity to manage their health risk (4, 14). When delivered in a participatory format combining lectures, discussions, and self-monitoring tools, they support behavioral ownership and health autonomy. Group-based education, especially among socially influential cohorts like teachers, enhances accountability and reinforces adherence to health behaviors through collective motivation. Although favorable effects on knowledge and behavior change following health education interventions are often reported, few studies have investigated direct improvements in teachers' preventive practices or metabolic improvement associated with type 2 diabetes (T2D). The current finding is critical, because it fills a gap in knowledge given the increasing diabetes burden in Iraq. Recent reports estimate that 13–14% of adults live with diabetes; rates continue to rise across the Kurdistan Region including Erbil (15). High school teachers, who play a major role modeling healthy behaviors to students and the community at large, represent an important target population for T2D prevention. Additionally, teachers have been exposed to occupational stress which may lead to prolonged sedentary life style habit, irregular lifestyle pattern and heavy work load than normal occupation that may increase risk of lifestyle disorders and unhealthy health behaviors. Thus, improving diabetes-related preventive behaviors among teachers may not only improve their health but also promote public health by raising awareness of common chronic disease risk factors and positively influencing behavior in school settings. Therefore, the present study aimed to evaluate the effect of a structured health education program on preventive practices and metabolic improvement related to T2D among high school teachers in Erbil, Iraq.

## 2 Research question

What is the effect of a structured health education program on preventive practices and metabolic improvement related to type 2 diabetes among high school teachers in Erbil City?

## 3 Methods

### 3.1 Study design, setting, period, and sampling

This study utilized a quasi-experimental design involving intervention and control groups and was conducted in Erbil, Iraq, across five major high schools. Participants were initially recruited through purposive sampling between October 27, 2024, and June 1, 2025, after which eligible participants were randomly allocated to the intervention and control groups using the lottery method.

### 3.2 Sample size

The sample size was determined using power analysis appropriate for a quasi-experimental intervention design. The calculation was based on detecting statistically significant changes in preventive practices and metabolic outcomes before and after the health education intervention, using a 5% significance level, 80% statistical power, and an expected moderate effect size. The minimum required sample size was estimated to be 197 participants. However, due to feasibility constraints, participant availability, and institutional access limitations during the study period, only 102 high school teachers were ultimately enrolled in the study. Therefore, the findings should be interpreted with caution, as the reduced sample size may have affected the statistical power and generalizability of the results.

### 3.3 Inclusion/exclusion

For participation in this study, both male and female teachers who were healthy from public schools were included as long as they had completed at least 1 year of work experience in the selected schools, regardless of type of contract (permanent, temporary or contractual). Teachers were targeted because they are well-positioned as role models in the school context and will help disseminate healthy lifestyle behaviors to students and communities at large. Moreover, the requirements and stress of being a teacher can render them vulnerable to their engagement in low-levels lifestyle habits which operate through metabolic pathways that are related with the onset of T2D. However, teachers who had type 2 diabetes or any of its complications in the past, those who had participated in a previous version of this program (or others based on academic performance improvement), those with other serious health conditions during the study period, teachers who were recruited for the pilot study and teachers released from that study to facilitate data collection were exclude.

### 3.4 Health education intervention

The health education program consisted of brief face-to-face educational sessions and followed by booklet-based self-learning (intervention period). The interventions were administered over eight weekly sessions (20–30 min) covering the following topics: healthy nutrition, physical activity and weight management, reduction of stress and awareness of HbA1c levels, hydration and reducing tobacco use as well as lifestyle modification for prevention and metabolic improvement. Sessions were delivered by a combination of certified health educators and doctoral-level educators, with support from a physician and dietitian using interactive teaching approaches including presentations, group discussion, question-and-answer sessions, and goal setting activities. In addition, participants were provided with a structured educational booklet (self-directed learning) which further reinforced the content of the intervention during the study period.

### 3.5 Study tools and data collection

The questionnaire was divided into two main parts. The first part gathered demographic data, including age group, gender, marital status, place of residence, years of teaching experience, teaching specialty, educational level, family history of diabetes, and smoking status. The second part included self-structured scale to assess practice regarding type 2 diabetes prevention and metabolic risk reduction. The questionnaire was provided in Kurdish, and any unclear questions were explained by the researchers. Data were collected by distributing questionnaires to participants who met the inclusion criteria. Each participant was allotted a total of 15–20 min to complete the questionnaire.

### 3.6 Pilot study

Before the main study, a pilot study was conducted to test clarity, feasibility and internal consistency of the self-structured preventive practices and metabolic improvements questionnaire. The pilot study was conducted between August 1st and September 15th, 2024, among 25 high school teachers who met the inclusion criteria but were not included in the final study sample. The purpose of the pilot testing was to evaluate the reliability and comprehensibility of the questionnaire items and to identify any ambiguities in wording or structure. Minor linguistic modifications were made to improve clarity following participant feedback. Internal consistency reliability was assessed using Cronbach's alpha coefficient. The overall Cronbach's alpha for the 15-item scale was calculated as 0.91, indicating an excellent level of internal consistency and reliability (16). According to established psychometric standards, a Cronbach's alpha value ≥ 0.90 reflects excellent reliability of the instrument. Data obtained from the pilot study were excluded from the final statistical analysis.

### 3.7 Measures

#### 3.7.1 Sociodemographic characteristics

The first section of the questionnaire included sociodemographic information of the teachers, such as date of birth, gender, specialty, years of teaching experience, type of school, and family history of diabetes.

#### 3.7.2 Preventive and metabolic improvement questionnaire

To assess the effectiveness of the health education program on lifestyle-related behaviors, a 15-item Preventive Practices and metabolic improvement Questionnaire was developed and administered. This instrument evaluated participants' adherence to evidence-based practices recommended for type 2 diabetes prevention and metabolic risk reduction. The items covered key domains including dietary choices (e.g., whole grains, healthy fats, avoiding processed foods), hydration, physical activity, stress management, smoking and alcohol avoidance, sleep hygiene, metabolic screening (e.g., HbA1c testing), and intermittent fasting. Each item was rated on a Likert scale from 1 (never) to 5 (always), with higher scores indicating stronger adherence to recommended practices. The scale's reliability was confirmed during a pilot study with a Cronbach's alpha of 0.87, indicating good internal consistency (16).

### 3.8 Ethical approval and inform consent

Ethical approval for this study was obtained from the College of Nursing Ethics Committee at Hawler Medical University, Erbil, Iraq (Ethics Approval No. 2432; Date: 22 August 2024). The study was conducted in accordance with the Declaration of Helsinki and institutional ethical guidelines. Written informed consent was obtained from all participants prior to participation in the study.

### 3.9 Statistical analysis

Data were summarized using frequencies and percentages for categorical variables (age group, gender, marital status, residence, years of experience, specialty, education level, family history of diabetes, and smoking status). Continuous variables, including preventive practice scores and clinical measurements (weight, BMI, waist circumference, blood pressure, fasting glucose, HbA1c, and lipid profile), were presented as mean ± standard deviation (SD). Baseline differences between intervention and control groups for categorical variables were assessed using the chi-square test. Within-group pre- and post-intervention comparisons were conducted using paired sample *t*-tests. Between-group comparisons of post-intervention outcomes were performed using independent sample *t*-tests where appropriate. One-way ANOVA was used to examine differences in practice score changes across demographic subgroups within the intervention group, followed by Tukey's HSD *post-hoc* tests for pairwise comparisons when significant differences were observed. Multiple linear regression analysis was conducted to identify independent predictors of practice score change, with group assignment and relevant demographic variables entered as predictors. Standardized beta coefficients (β), 95% confidence intervals (CI), and *p*-values were reported. Effect sizes were calculated using Cohen's d to determine the magnitude of intervention effects. Effect size interpretation followed Cohen's criteria: small (0.20–0.49), medium (0.50–0.79), and large (≥ 0.80). All statistical analyses were performed using SPSS version 27 (IBM Corp., Armonk, NY, USA). A two-tailed *p*-value < 0.05 was considered statistically significant.

## 4 Results

### 4.1 Socio-demographic characteristics of the study participants

A total of 102 high school teachers participated in the study, with 51 assigned to the intervention group and 51 to the control group. The mean age was slightly higher in the intervention group (45.16 ± 8.86 years) than in the control group (43.43 ± 9.08 years). The largest age subgroup in both arms was 42–49 years, accounting for 17 (33.3%) in the intervention and 16 (31.4%) in the control group. Statistically significant differences were observed across several baseline characteristics. Notably, marital status differed, with 6 (11.8%) single participants in the intervention group versus 4 (7.8%) in the control (*p* = 0.03). All participants in the intervention group were urban residents (100%), compared to 47 (92.2%) in the control group, with 4 (7.8%) from the countryside (*p* = 0.02). Additionally, smoking status was significantly different, as 14 (27.5%) of control group participants were smokers versus only 5 (9.8%) in the intervention group (*p* < .01). A significant difference was also noted in educational level, with 3 (5.9%) diploma holders in the intervention group and 2 (3.9%) postgraduates in the control group (*p* = 0.02). Detailed demographics and other variables are presented in Table 1.

**Table 1:** Socio-demographic characteristics of the study sample (51 samples per group).

Variable	Type of variable	Group classification		*P*-value
		Intervention group No. (%)	Control group No. (%)	
Age group	18–25	2 (3.90)	1 (2.00)	0.03
	26–33	3 (5.90)	8 (15.70)	
	34–41	12 (23.50)	12 (23.50)	
	42–49	17 (33.30)	16 (31.40)	
	50 and above	17 (33.30)	14 (27.50)	
	Mean ± SD	45.16 ± 8.86	43.43 ± 9.08	
Gender	Male	25 (49.00)	28 (54.90)	0.40
	Female	26 (51.00)	23 (45.10)	
Marital status	Single	6 (11.80)	4 (7.80)	0.03
	Married	42 (82.40)	46 (90.20)	
	Divorced/Widowed	3 (5.90)	1 (2.00)	
Residence	City	51 (100.00)	47 (92.20)	0.02
	Countryside	–	4 (7.80)	
Years of experience	5–10 years	6 (11.80)	11 (21.60)	0.15
	11–15 years	3 (5.90)	3 (5.90)	
	15 and above	42 (82.40)	37 (72.50)	
Teacher specialty	Linguistics	21 (41.20)	14 (27.50)	0.08
	Mathematics	7 (13.70)	9 (17.60)	
	Science	12 (23.50)	18 (35.30)	
	Social	6 (11.80)	8 (15.70)	
	Art	3 (5.90)	1 (2.00)	
	Sports	2 (3.90)	1 (2.00)	
Educational level	Diploma	3 (5.90)	–	0.02
	Bachelor's Degree	48 (94.10)	49 (96.10)	
	Master's/PhD	–	2 (3.90)	
Family history of diabetes	Yes	27 (52.90)	22 (43.10)	0.33
	No	24 (47.10)	29 (56.90)	
Do you smoke?	Yes	5 (9.80)	14 (27.50)	< 0.01
	No	46 (90.20)	37 (72.50)	

Chi-square test was used for categorical comparisons. “–” indicates no participants in that category. A p-value < 0.05 was considered statistically significant. Significant differences were observed in age, marital status, residence, education level, and smoking status.

### 4.2 Changes in preventive practices following the health education program

The results showed that the health education program significantly improved multiple preventive practices and metabolic improvement among high school teachers. The overall practice score increased notably from 45.08 at pre-test to 59.58 at post-test, indicating broad behavioral enhancement. The most substantial improvements were observed in HbA1c testing (from 1.86 to 3.75, *p* < .001), label reading for sugar content (2.59 to 3.96, *p* < .001), and preference for water or natural juices over sugary drinks (3.08 to 4.20, *p* < .001). Other critical gains included regular intermittent fasting (2.41 to 3.59, *p* < .001), increased physical activity (2.59 to 3.43, *p* < .001), and healthier food choices like including lean proteins (2.94 to 3.84, *p* < .001). The only non-significant change was in smoking avoidance (*p* = 0.158), possibly due to already high baseline scores. For further details, see Table 2.

**Table 2:** Comparison of teachers' preventive practices related to type 2 diabetes before and after the health education program (*n* = 51).

Practice item	Pre–test mean	Post–test mean	*t*–value	*P*–value
Prefer whole grains over refined grains	2.41	4.69	2.22	0.029
Choose water or natural juices over sugary beverages	3.08	4.20	6.86	< 0.001
Include lean proteins and healthy fats in meals	2.94	3.84	5.63	< 0.001
Avoid processed and fried foods high in sugar, salt, and unhealthy fats	3.04	3.94	5.07	< 0.001
Read food labels to monitor sugar and ingredients	2.59	3.96	6.72	< 0.001
Eat out more often than home-cooked meals	2.51	2.14	−2.30	0.023
Engage in ≥150 mins moderate or ≥75 mins vigorous weekly physical activity	2.59	3.43	4.28	< 0.001
Maintain healthy weight through diet and exercise	2.80	3.82	6.27	< 0.001
Drink at least 8 glasses of water per day	2.88	4.00	6.11	< 0.001
Use stress reduction strategies (e.g., meditation, yoga, deep breathing)	2.45	3.57	5.31	< 0.001
Get 7–8 h of sleep nightly	3.49	4.22	3.88	< 0.001
Avoid smoking or similar harmful habits	4.39	4.69	1.42	0.158
Avoid alcohol consumption	4.14	4.84	3.33	0.001
Practice intermittent fasting regularly	2.41	3.59	5.45	< 0.001
Have undergone HbA1c testing	1.86	3.75	8.48	< 0.001
Overall practice score (sum of all items)	**45.08**	**59.58**	—	—

All comparisons are based on paired sample t-tests. Statistically significant results are indicated at P < 0.05. Higher scores reflect stronger adherence to recommended practices for Type 2 diabetes prevention and metabolic risk reduction.

### 4.3 Preventive practice trends in the control group

The results revealed that the control group did not exhibit any statistically significant changes in preventive practices or metabolic outcomes related to type 2 diabetes from pre-test to post-test. All practice items showed minimal differences, with *p*-values ranging from 0.677 to 0.929, indicating no meaningful behavioral shifts. For instance, scores for drinking at least 8 glasses of water daily changed only slightly from 2.86 to 2.94 (*p* = 0.677), and practicing intermittent fasting remained nearly the same (2.43 to 2.37, *p* = 0.798). Likewise, the overall practice score showed a negligible decline from 44.54 to 44.35, reinforcing the lack of impact in the absence of the educational intervention. For more details, refer to Table 3.

**Table 3:** Comparison of the control group's practices related to type 2 diabetes prevention and metabolic risk reduction (Pre- vs Post-Test).

Practice item	Pre-test mean	Post-test mean	*t*-value	*P*-value
Prefer whole grains over refined grains	2.39	2.45	0.34	0.736
Choose water or natural juices over sugary beverages	3.06	3.12	0.28	0.782
Include lean proteins and healthy fats in meals	2.92	2.88	−0.23	0.821
Avoid processed and fried foods high in sugar, salt, and unhealthy fats	3.02	2.96	−0.41	0.685
Read food labels to monitor sugar and ingredients	2.57	2.53	−0.19	0.853
Eat out more often than home-cooked meals	2.55	2.59	0.18	0.857
Engage in ≥150 mins moderate or ≥75 mins vigorous weekly physical activity	2.61	2.55	−0.31	0.759
Maintain healthy weight through diet and exercise	2.78	2.73	−0.27	0.791
Drink at least 8 glasses of water per day	2.86	2.94	0.42	0.677
Use stress reduction strategies (e.g., meditation, yoga, deep breathing)	2.47	2.41	−0.29	0.774
Get 7–8 h of sleep nightly	3.51	3.47	−0.21	0.836
Avoid smoking or similar harmful habits	4.37	4.35	−0.09	0.929
Avoid alcohol consumption	4.12	4.08	−0.18	0.858
Practice intermittent fasting regularly	2.43	2.37	−0.26	0.798
Have undergone HbA1c testing	1.88	1.92	0.15	0.881
Overall practice score (sum of all items)	**44.54**	**44.35**	—	—

All comparisons are based on paired sample t-tests. Statistically significant results are indicated at P < 0.05. Higher scores reflect stronger adherence to recommended practices for Type 2 diabetes prevention and metabolic risk reduction.

### 4.4 Impact of the health education program on clinical and anthropometric outcomes

The results demonstrated significant improvements in all measured clinical, anthropometric, and metabolic indicators among participants in the intervention group following implementation of the health education program. Statistically significant reductions were observed in body weight, decreasing from 78.4 ± 12.6 kg at baseline to 75.8 ± 12.1 kg at follow-up (*p* = 0.002), accompanied by corresponding decreases in BMI (28.7 to 27.8 kg/m^2^, *p* = 0.008) and waist circumference (94.3 to 91.2 cm, *p* < .001). Cardiovascular parameters also improved, including reductions in systolic blood pressure (128.6 to 124.2 mmHg, *p* = 0.012) and diastolic blood pressure (82.4 to 79.8 mmHg, *p* = 0.018). Significant metabolic indicator improvements were observed in fasting blood glucose (98.7 to 94.3 mg/dl, *p* = 0.004) and HbA1c levels (5.8% to 5.5%, *p* < .001). In addition, participants demonstrated favorable changes in lipid profile parameters, including reductions in total cholesterol (198.4 to 185.7 mg/dl, *p* = 0.001), LDL cholesterol (124.8 to 115.3 mg/dl, *p* = 0.003), and triglycerides (156.3 to 142.8 mg/dl, *p* = 0.006), along with a significant increase in HDL cholesterol from 48.2 to 52.4 mg/dl (*p* < .001). Further details are presented in Table 4.

**Table 4:** Clinical and anthropometric measurements pre- and post-intervention (intervention group, *n* = 51).

Measurement	Baseline mean ±SD	Follow-up mean ±SD	*P*-value
Weight (kg)	78.4 ± 12.6	75.8 ± 12.1	0.002
BMI (kg/m^2^)	28.7 ± 4.2	27.8 ± 4.0	0.008
Waist Circumference (cm)	94.3 ± 8.9	91.2 ± 8.4	< 0.001
Waist-to-Hip Ratio	0.89 ± 0.07	0.86 ± 0.06	0.001
Systolic BP (mmHg)	128.6 ± 14.3	124.2 ± 12.8	0.012
Diastolic BP (mmHg)	82.4 ± 9.2	79.8 ± 8.6	0.018
Fasting Glucose (mg/dl)	98.7 ± 11.4	94.3 ± 9.8	0.004
HbA1c (%)	5.8 ± 0.4	5.5 ± 0.3	< 0.001
Total Cholesterol (mg/dl)	198.4 ± 28.3	185.7 ± 24.9	0.001
LDL Cholesterol (mg/dl)	124.8 ± 22.7	115.3 ± 20.1	0.003
HDL Cholesterol (mg/dl)	48.2 ± 8.6	52.4 ± 9.2	< 0.001
Triglycerides (mg/dl)	156.3 ± 34.7	142.8 ± 29.4	0.006

Data are presented as mean ± standard deviation, and P-values were calculated using paired sample t-tests. Bold values indicate statistical significance (P < 0.05).

BP, Blood Pressure; BMI, Body Mass Index; HbA1c, Glycated Hemoglobin; LDL, Low-Density Lipoprotein; HDL, High-Density Lipoprotein.

### 4.5 Effect sizes for preventive practices and clinical outcomes

The study findings indicated that the health education program had the ability to markedly improve preventive practices and metabolic indicators among high school teachers. A very large between-group effect size was observed for the overall practice score (*d* = 1.94), confirming the program's strong behavioral impact. Several individual practices also showed large effects, including HbA1c testing (*d* = 1.17), preference for whole grains (*d* = 1.15), reading food labels (*d* = 0.95), and replacing sugary beverages with healthier options (*d* = 0.98). Medium to large effects were noted for intermittent fasting (*d* = 0.78) and stress reduction (*d* = 0.76). In terms of clinical outcomes, the strongest effect was observed in HbA1c reduction (*d* = 0.86, large), followed by small-to-medium effects in fasting glucose (*d* = 0.45), HDL cholesterol (*d* = 0.46), and waist-to-hip ratio (*d* = 0.45) (Table 5).

**Table 5:** Effect sizes for preventive practices and clinical measurements.

Domain	Variable	Intervention d	Control d	Between-group d	Interpretation
Preventive practices	Overall practice score	1.89	0.02	1.94	Very large
	Prefer whole grains over refined grains	1.12	0.03	1.15	Large
	Choose water/natural juices over sugary beverages	0.96	0.02	0.98	Large
	Include lean proteins and healthy fats	0.78	−0.02	0.80	Large
	Avoid processed and fried foods	0.71	−0.03	0.74	Medium–large
	Read food labels for sugar content	0.94	−0.01	0.95	Large
	Engage in adequate physical activity	0.59	−0.02	0.61	Medium
	Maintain healthy weight	0.87	−0.02	0.89	Large
	Drink adequate water daily	0.85	0.04	0.81	Large
	Use stress reduction strategies	0.74	−0.02	0.76	Medium–large
	Get adequate sleep	0.54	−0.01	0.55	Medium
	Practice intermittent fasting	0.76	−0.02	0.78	Medium–large
	Undergo HbA1c testing	1.18	0.01	1.17	Large
Clinical measurements	Weight (kg)	0.41	−0.01	0.42	Small–medium
	BMI (kg/m^2^)	0.22	−0.01	0.23	Small
	Waist Circumference (cm)	0.36	−0.03	0.39	Small–medium
	Waist-to-Hip Ratio	0.45	0.00	0.45	Small–medium
	Systolic Blood Pressure	0.32	−0.01	0.33	Small–medium
	Diastolic Blood Pressure	0.29	−0.01	0.30	Small
	Fasting Glucose (mg/dl)	0.42	−0.03	0.45	Small–medium
	HbA1c (%)	0.86	0.00	0.86	Large
	Total Cholesterol (mg/dl)	0.47	−0.02	0.49	Small–medium
	LDL Cholesterol (mg/dl)	0.44	−0.01	0.45	Small–medium
	HDL Cholesterol (mg/dl)	0.47	0.01	0.46	Small–medium
	Triglycerides (mg/dl)	0.42	−0.02	0.44	Small–medium

Effect sizes were calculated using Cohen's d formula [(Mean1 – Mean_2_) / pooled standard deviation], where within-group values represent pre- to post-intervention changes and between-group values compare post-intervention differences. According to Cohen's conventions, d = 0.20–0.49 indicates a small effect, 0.50–0.79 a medium effect, 0.80–1.19 a large effect, and ≥1.20 a very large effect. Bolded values in the table highlight medium to very large effects (d ≥ 0.50).

### 4.6 Differences in practice score improvements across demographic subgroups

The results revealed that several demographic factors significantly influenced the degree of improvement in preventive practice scores following the intervention. Age group showed a significant association (*p* = 0.025, η^2^ = 0.18), with the 18–33 age group demonstrating the highest mean score change (18.2 ± 3.8) compared to older age groups, particularly those aged 50 and above (12.8 ± 4.1). A strong effect was also observed based on teacher specialty (*p* = 0.004, η^2^ = 0.30), where linguistics teachers achieved the greatest improvement (16.2 ± 3.9), while sports/PE teachers showed the least (9.5 ± 2.1). Most notably, baseline practice levels had the strongest effect (*p* < .001, η^2^ = 0.39), with participants in the lowest tertile (< 42 points) achieving the greatest gain (18.8 ± 3.2) compared to those in the highest tertile (>48 points), who improved less (10.6 ± 3.8) Table 6.

**Table 6:** ANOVA comparison of changes in preventive practice scores across demographic subgroups (Intervention Group, *n* = 51).

Demographic variable	Subgroup	*n*	Practice score change (Mean ±SD)	Test statistic	*P*-value	Effect size
Age group	18–33 years	5	18.2 ± 3.8^a^	F = 3.42	0.025	η^2^ = 0.18
	34–41 years	12	16.4 ± 4.2^ab^			
	42–49 years	17	14.1 ± 3.6^b^			
	50+ years	17	12.8 ± 4.1^b^			
Gender	Male	25	12.9 ± 3.8	*t* = 5.87	0.19	Cohen's d = 0.11
	Female	26	16.1 ± 4.2			
Years of experience	5–10 years	6	17.3 ± 4.1	F = 2.89	0.66	η^2^ = 0.11
	11–15 years	3	15.7 ± 2.9			
	≥15 years	42	14.0 ± 4.0			
Teacher specialty	Linguistics	21	16.2 ± 3.9^a^	F = 4.16	0.004	η^2^ = 0.30
	Mathematics	7	15.8 ± 3.2^ab^			
	Science	12	14.1 ± 4.1^ab^			
	Social Studies	6	11.5 ± 3.6^b^			
	Art	3	12.3 ± 2.8^ab^			
	Sports/PE	2	9.5 ± 2.1^b^			
Education level	Diploma	3	11.0 ± 2.6^a^	F = 6.23	0.40	η^2^ = 0.20
	Bachelor's Degree	48	14.8 ± 4.0^b^			
	Master's/PhD	—	—			
Family history of diabetes	Yes	27	16.4 ± 3.7	*t* = 8.94	0.42	Cohen's d = 0.15
	No	24	12.4 ± 3.9			
Smoking status	Smoker	5	9.8 ± 3.2	*t* = 12.45	0.10	Cohen's d = 0.20
	Non-smoker	46	15.2 ± 3.8			
Baseline practice score (Tertiles)	Low (< 42 points)	17	18.8 ± 3.2^a^	F = 15.67	< 0.001	η^2^ = 0.39
	Medium (42–48 points)	17	14.2 ± 3.1^b^			
	High (> 48 points)	17	10.6 ± 3.8^c^			

Thus, practice score change was determined by subtracting pre- from post-intervention scores. One-way ANOVA was used for outcome variables with three or more categories; Independent samples Student's t-test was used for those with two categories. Uppercase letters (A, B) indicate statistically significant between group comparisons; superscript letters (^a^, ^b^, ^c^) indicate where the key statistical differences were identified in subgroups. Statistical significance is denoted by bold p -values (p < 0.05). “—” means that there were zero participants in that category.

### 4.7 *Post-Hoc* comparisons of practice score changes across demographic subgroups

The results revealed significant pairwise differences in preventive practice score improvements across age groups, teacher specialties, and baseline practice tertiles. Among age groups, participants aged 18–33 years showed significantly greater improvements than those aged 50 and above (mean difference = 5.4, *p* = 0.019), and those aged 34–41 years also improved more than the oldest group (mean difference = 3.6, *p* = 0.041). Regarding teacher specialty, linguistics teachers had significantly higher improvements than those in social studies (mean difference = 4.7, *p* = 0.012) and sports/PE (mean difference = 6.7, *p* = 0.008). Additionally, mathematics and science teachers both outperformed sports/PE teachers in practice score gains (*p* = 0.043 and *p* = 0.045, respectively). The strongest and most consistent differences were observed across baseline practice tertiles, with the low baseline group showing significantly greater improvements compared to both the medium (mean difference = 4.6, *p* < .001) and high baseline group (mean difference = 8.2, *p* < .001), while the medium group also outperformed the high group (*p* = 0.003). For more details, refer to Table 7.

**Table 7:** Tukey's HSD *Post-hoc* pairwise comparisons for practice score changes.

Comparison	Mean difference	95% CI	*P*-value
**Age group**	
18–33 vs. 34–41 years	1.8	−2.4–6.0	0.664
18–33 vs. 42–49 years	4.1	−0.3–8.5	0.078
18–33 vs. 50+ years	5.4	1.0–9.8	0.019
34–41 vs. 42–49 years	2.3	−1.2–5.8	0.312
34–41 vs. 50+ years	3.6	0.1–7.1	0.041
42–49 vs. 50+ years	1.3	−1.8–4.4	0.687
**Teacher specialty**	
Linguistics vs. Social Studies	4.7	1.2–8.2	0.012
Linguistics vs. Sports/PE	6.7	2.1–11.3	0.008
Linguistics vs. Mathematics	0.4	−3.8–4.6	0.995
Linguistics vs. Science	2.1	−1.4–5.6	0.456
Linguistics vs. Art	3.9	−2.1–9.9	0.345
Mathematics vs. Social Studies	4.3	−0.8–9.4	0.134
Mathematics vs. Sports/PE	6.3	0.2–12.4	0.043
Mathematics vs. Science	1.7	−2.8–6.2	0.789
Mathematics vs. Art	3.5	−3.7–10.7	0.624
Science vs. Sports/PE	4.6	0.1–9.1	0.045
Science vs. Social Studies	2.6	−1.9–7.1	0.458
Science vs. Art	1.8	−4.8–8.4	0.925
Social Studies vs. Sports/PE	2.0	−4.1–8.1	0.867
Social Studies vs. Art	−0.8	−8.2–6.6	0.989
Art vs. Sports/PE	2.8	−5.4–11.0	0.845
**Baseline Score Tertiles**	
Low vs. Medium	4.6	2.1–7.1	< 0.001
Low vs. High	8.2	5.7–10.7	< 0.001
Medium vs. High	3.6	1.1–6.1	0.003

P-values were considered statistically significant at p < 0.05.

CI, Confidence Interval.

### 4.8 Predictors of post-intervention preventive practice scores

The results revealed that group assignment was the strongest predictor of post-intervention practice scores, with participants in the intervention group scoring significantly higher than those in the control group (B = 12.43, *p* < .001, 95% CI: 10.09 to 14.77). Among demographic factors, younger age was associated with higher practice scores (B = −0.18, *p* = 0.012), indicating that each additional year of age slightly reduced expected score gains. Female participants also had significantly higher scores compared to males (B = 2.84, *p* = 0.010), and those with a family history of diabetes showed improved scores (B = 2.17, *p* = 0.029). Conversely, smoking status was negatively associated with practice outcomes, with smokers scoring 3.76 points lower than non-smokers (*p* = 0.011). Years of experience and educational level did not significantly predict changes in practice scores. For extra details, refer to Table 8.

**Table 8:** Multiple linear regression analysis predicting post-intervention practice scores (*N* = 102).

Predictor variable	B	SE	β	*t*	*P*-value	95% CI (Lower, Upper)
(Constant)	12.84	3.67	—	3.50	< 0.001	5.58, 20.10
Group assignment (Intervention = 1)	12.43	1.18	0.45	10.54	< 0.001	10.09, 14.77
Age (years)	−0.18	0.07	−0.12	−2.57	0.012	−0.32,−0.04
Gender (Female = 1)	2.84	1.09	0.11	2.61	0.010	0.68, 5.00
Family history of diabetes (Yes = 1)	2.17	0.98	0.09	2.21	0.029	0.22, 4.12
Smoking status (Smoker = 1)	−3.76	1.45	−0.11	−2.59	0.011	−6.64,−0.88
Years of experience	0.09	0.08	0.06	1.13	0.262	−0.07, 0.25
Education level (Bachelor's = 1)	1.82	1.52	0.05	1.20	0.233	−1.19, 4.83

Practice score change was the dependent variable. B, unstandardized coefficient; SE, standard error; β, standardized beta; CI, confidence interval; Adjusted R^2^, variance explained by the model. P < 0.05 was considered significant.

## 5 Discussion

This study is one of the first to evaluate a structured health education intervention's influence on both preventive practice and metabolic improvement related to type 2 diabetes, among high school teachers of Erbil as far as we know. Our findings show that the program led to significant improvements in lifestyle behaviors and practice scores across multiple domains. The overall practice scores increased by 32% in the intervention group as compared to the control; healthy beverage and choice behavior increased by 50%; HbA1c-testing behavior and monitoring practices increased by 27%. Improvements were also observed in intermittent fasting, label reading, lean protein consumption, physical activity, and water intake. By domain, the largest improvements were seen in blood glucose management and dietary awareness, suggesting strong program impact. According to the effect size estimates, several behaviors such as label reading, HbA1c testing, and grain choices achieved large to very large magnitudes of change. These trends were not observed in the control group, which showed negligible changes and even a small decline in the composite practice score.

The educational program appeared effective in improving preventive practices and metabolic-related behaviors among teachers by enhancing health literacy, increasing awareness of lifestyle-related risk factors, and encouraging sustainable behavior change within the school environment. The community-based and interactive nature of the intervention may have strengthened participant engagement, peer support, and social modeling, thereby reinforcing healthy lifestyle behaviors. In addition, the use of culturally appropriate educational approaches may have improved participant understanding, motivation, and long-term adherence to preventive health practices. Behavioral science principles integrated into the program may also have contributed to greater self-efficacy and behavioral ownership among participants.

Thus, this reinforces the notion that program wide influences extend beyond didactic elements of preventive and metabolic improvement behaviors to psychosocial engagement. Teachers not only absorbed the content but internalized its relevance, sharing what they learned with colleagues and families. For instance, participants reported feeling more empowered to discuss healthy eating in staff rooms and more mindful of their food choices during lunch breaks. As part of the working of diffusion, informal sharing of health-related knowledge among participants would have contributed to maintain the established advantages from behavioral change and metabolic improvements. In fact, simulated peer support, social modeling and accountability mechanisms integrated into health education programs can improve the effectiveness of interventions. In a recent review, peer-supported diabetes self-management interventions were associated with glycemic benefit in more than two-thirds of included studies; greater reduction in HbA1c (up to 2.7%) and improved long-term adherence to healthy diet regimens and lifestyle behaviors over follow-up periods between 6 and 12 months (17). Teachers in our study who actively discussed their progress in groups also showed higher post-test practice scores. Moreover, participants who shared printed handouts with their families or students reported a greater sense of commitment to the new behaviors. Taken together, these elements reflect a holistic approach that targets both individual change and broader environmental support.

Previous studies assessing school-based interventions for metabolic disorders among adults have yielded encouraging but mixed outcomes (18, 19). Some focused solely on nutrition education, while others combined exercise promotion with counseling, often reporting moderate practice improvements (20). In Iraq, few structured programs have targeted non-health professionals for diabetes prevention, especially using behavioral strategies. In 2013, a pilot study among Kurdish municipal workers showed a rise in practice scores but lacked statistical power (21). A similar study conducted in Turkey involving female teachers also led to an increase of fruit and vegetable intake (but not glycemic screening in 202 (22). Multifaceted educational programmes were shown to be more effective than usual care in reducing BMI and HbA1c levels and encouraging diabetes screening behavior (22, 23), which corresponds with the conclusions of this study, in studies from India, Egypt and Iran published around 2010. A cluster-RCT in Bangladesh reported a 28% increase in dietary practice adherence following teacher training (24). While most literature focuses on student-focused interventions, emerging data support targeting educators as behavior multipliers within communities. Our results strengthen this argument and highlight the untapped potential of using teachers in non-communicable disease prevention strategies. As role models, educators can normalize lifestyle changes in the school setting, amplifying the public health impact.

The results of our study spanning behavioral, metabolic, and demographic dimensions demonstrate alignment with the growing evidence supporting targeted adult education in school settings. However, our effect sizes are generally larger, likely due to high baseline engagement, multi-pronged delivery, and contextual tailoring. The integration of visual materials, real-life examples, and community-relevant illustrations may have helped improve relevance and retention. Participants also expressed strong satisfaction with the session structure, reinforcing the relationship between positive learning environments and sustained behavior change. Based on post-program feedback, many participants recommended extending similar programs to parents, school cafeterias, and educational administrators, highlighting opportunities for broader implementation. These findings suggest the potential sustainability of the intervention and its possible long-term benefits if supported by periodic reinforcement sessions. However, the absence of booster sessions may limit the long-term maintenance of certain behavioral changes, particularly among high-risk individuals. These patterns are consistent with the literature on habit formation, which notes that reinforcement and environmental cues are critical for maintaining lifestyle changes. Future studies could explore the effect of low-cost reinforcement tools like text reminders, posters in school corridors, or peer mentoring to extend impact.

However, scaling up such programs may face barriers if institutional support, budget allocations, or training logistics are lacking. Administrative endorsement is key to ensuring integration into professional development systems and minimizing attrition. Additionally, health promotion materials should be updated regularly to reflect evolving scientific guidelines. For example, recent guidelines suggest incorporating stress-reduction strategies into diabetes prevention frameworks (25). Despite enthusiasm among participating teachers, some reported time constraints and curriculum overload, pointing to the need for simplified formats or modular delivery. Context-sensitive adaptation especially for rural schools will be vital in future implementations.

In our study, younger teachers and those from humanities backgrounds showed the greatest behavior gains, indicating the relevance of demographic tailoring. For instance, teachers under 34 years of age improved their scores by an average of 5.4 points more than those over 50. Likewise, teachers with baseline low practice scores saw nearly twice the improvement compared to high-score peers. These patterns echo findings from diabetes education programs in other LMICs, where age, specialty, and readiness to change modulated program effectiveness (26, 27). Our regression analysis further reinforced these findings, with baseline score, group assignment, and gender as the strongest predictors of post-test performance. This highlights the importance of stratifying content or delivery methods based on key participant characteristics. This underscores the need to stratify based on specific characteristics of participants with evidence. Lastly, this study has several limitations. Variations in participant characteristics, school environments, lifestyle factors, and the reduced final sample size may have contributed to differences between groups at baseline. Therefore, the findings should be interpreted with caution, as these factors may have influenced the internal validity and comparability of the study outcomes. Additionally, the relatively short follow-up duration may not have fully captured long-term behavioral retention. The use of purposive sampling and the inclusion of only urban high school teachers in Erbil may limit the representativeness and generalizability of the findings. In addition, the absence of blinding and reliance on self-reported data may have introduced performance, recall, and social desirability biases. Another important limitation is that the final analyzed sample size (*N* = 102) was smaller than the initially estimated sample size (N = 197), which may have reduced the statistical power and generalizability of the study. This discrepancy was primarily related to feasibility and logistical constraints during intervention implementation. Furthermore, objective measures of long-term adherence to lifestyle modifications were not assessed. Nevertheless, statistically significant improvements were observed across several preventive and metabolic outcome measures. Future studies with larger and more diverse samples and longer follow-up periods are recommended to confirm and extend these findings.

## 6 Conclusion

The structured health education program significantly enhanced preventive practices and metabolic health, resulting in meaningful improvements in clinical and behavioral outcomes among high school teachers in Erbil. These findings underscore the critical role of targeted educational interventions in promoting health literacy and facilitating sustainable lifestyle changes to prevent type 2 diabetes. Policymakers and healthcare authorities are encouraged to integrate such programs into school health strategies to create a ripple effect across communities. Future initiatives should explore long-term follow-up, digital delivery methods, and the expansion of similar programs to other professional groups and younger populations to maximize impact and sustainability.

## Data Availability

The data analyzed in this study is subject to the following licenses/restrictions: No Restriction. Requests to access these datasets should be directed to Shalawahmed997@gmail.com.
